# Medulloblastoma in Adolescents and Young Adults (AYA): Bridging Pediatric Paradigms and Adult Oncology Practice

**DOI:** 10.3390/jcm14134472

**Published:** 2025-06-24

**Authors:** Antonio Ruggiero, Giorgio Attinà, Dario Talloa, Stefano Mastrangelo, Alberto Romano, Palma Maurizi, Silvia Chiesa, Gianpiero Tamburrini, Alessandro Olivi, Alessio Albanese

**Affiliations:** 1 Pediatric Oncology Unit, Fondazione Policlinico Universitario Agostino Gemelli IRCCS, 00168 Rome, Italy; giorgio.attina@policlinicogemelli.it (G.A.); dario.talloa@guest.policlinicogemelli.it (D.T.); stefano.mastrangelo@unicatt.it (S.M.); alberto.romano@guest.policlinicogemelli.it (A.R.); palma.maurizi@unicatt.it (P.M.); 2 Department of Woman and Child Health and Public Health, Università Cattolica del Sacro Cuore, 00168 Rome, Italy; 3 Gemelli Advanced Radiotherapy Center, Fondazione Policlinico Universitario Agostino Gemelli IRCCS, 00168 Rome, Italy; silvia.chiesa@policlinicogemelli.it; 4 Pediatric Neurosurgery, Fondazione Policlinico Universitario Agostino Gemelli IRCCS, 00168 Rome, Italy; gianpiero.tamburrini@unicatt.it; 5 Department of Neuroscience, Section of Neurosurgery, Università Cattolica del Sacro Cuore, 00168 Rome, Italy; 6 Neurosurgery Unit, Department of Neurosciences, Fondazione Policlinico Universitario Agostino Gemelli IRCCS, 00168 Rome, Italy; alessandro.olivi@unicatt.it (A.O.); alessio.albanese@unicatt.it (A.A.); 7 Neurosurgery Unit, Department of Neurosciences, Università Cattolica del Sacro Cuore, 00168 Rome, Italy

**Keywords:** medulloblastoma, craniospinal irradiation, chemotherapy, survivorship care, adolescents, young adults

## Abstract

Medulloblastoma represents a rare yet complex embryonal tumor of the posterior cranial fossa that, while predominantly affecting pediatric populations, occurs with increasing recognition among adolescents and young adults (AYAs, 15–39 years). The scarcity of medulloblastoma within this demographic creates substantial obstacles in diagnosis, treatment selection, and psychosocial management that differ markedly from established pediatric approaches. Emerging data reveal that AYA patients exhibit distinctive tumor biology, including altered molecular subgroup patterns, variable therapeutic responses, and unique survival trajectories when compared to younger patients. Current investigations examining autologous stem cell transplantation following intensive chemotherapy protocols in metastatic cases demonstrate encouraging preliminary results. Evidence increasingly supports adapting pediatric treatment paradigms for adult application, potentially improving therapeutic outcomes while reducing treatment burden. These cross-disciplinary approaches between pediatric and adult oncology demonstrate considerable promise for enhancing clinical results and minimizing therapy-associated morbidity, emphasizing the critical need for collaborative care models in managing this challenging malignancy across diverse age groups.

## 1. Introduction

The management of medulloblastoma in adolescents and young adults (AYA) presents formidable challenges within contemporary neuro-oncology practice. Originally characterized by Cushing and Bailey nearly a century ago, this embryonal neoplasm of the cerebellar region creates distinct clinical scenarios when diagnosed in patients between 15 and 39 years of age [1,2]. Although accounting for fewer than 1% of adult central nervous system malignancies, medulloblastoma treatment in AYAs requires sophisticated integration of pediatric and adult oncological expertise [2,3,4]. This comprehensive analysis explores current therapeutic strategies, clinical obstacles, molecular discoveries, and psychosocial considerations specific to AYA medulloblastoma management.

## 2. Epidemiological Characteristics and Tumor Biology

Medulloblastoma demonstrates significant geographical and demographic variations in incidence patterns worldwide. Global epidemiological studies reveal an annual incidence ranging from 0.6 to 1.9 cases per 100,000 population in pediatric cohorts, with substantial regional disparities. North American registries report slightly higher incidence rates (1.5–1.9 per 100,000) compared to European populations (0.8–1.2 per 100,000), while Asian populations demonstrate intermediate rates (1.0–1.4 per 100,000) [5].

These variations may reflect genetic susceptibility differences, environmental factors, or diagnostic reporting disparities across healthcare systems.

Among AYA populations specifically, medulloblastoma incidence decreases dramatically to 0.05–0.1 cases per 100,000 population annually, with minimal regional variation observed globally. The AYA demographic comprises less than 1% of all CNS neoplasms in this age group across all studied populations [6,7]. Importantly, survival outcomes demonstrate regional disparities, with five-year overall survival rates ranging from 60 to 75% in resource-limited settings to 80–90% in high-income countries, primarily attributed to differences in access to specialized neurosurgical care, advanced radiotherapy techniques, and comprehensive multidisciplinary management [8].

Gender distribution patterns show consistent male predominance across all regions (male/female ratio 1.5–2:1), though this disparity becomes less pronounced in AYA populations compared to pediatric cohorts. Ethnic and racial variations have been documented, with slightly higher incidence rates reported in caucasian populations compared to African or Hispanic demographics, though these differences may reflect healthcare access disparities rather than true biological variation [9].

AYA medulloblastoma displays significant biological divergence from pediatric variants, encompassing altered molecular subgroup distributions, distinct therapeutic sensitivities, and divergent prognostic patterns [10]. These fundamental differences highlight the necessity for dedicated AYA-focused research initiatives. The tumor typically arises within cerebellar tissue and frequently demonstrates leptomeningeal dissemination at initial presentation.

Contemporary classification systems recognize four principal molecular subgroups with distinct age-dependent distributions: WNT-activated tumors occur in 10–15% of pediatric cases but only 8–12% of AYA cases, maintaining excellent prognosis (>95% overall survival) across all age groups [11,12]; SHH-activated tumors show marked age preference, representing 15–20% of pediatric cases but 40–60% of AYA cases, with AYA SHH tumors frequently harboring PTCH1 mutations (65% vs. 35% in pediatric cases) and demonstrating enhanced responsiveness to hedgehog pathway inhibitors; Group 3 tumors predominate in pediatric populations (25–30%) but decrease significantly in AYAs (10–15%), demonstrating high-risk features including MYC amplification; and Group 4 tumors represent the most common pediatric subtype (35–40%) but occur less frequently in AYAs (20–25%), characterized by chromosome 17 abnormalities and intermediate prognosis [13].

AYA tumors demonstrate unique molecular characteristics including intermediate mutational burden (median 0.8 mutations/Mb) compared to pediatric (0.5 mutations/Mb) and adult cases (1.2 mutations/Mb). DNA methylation patterns in AYA SHH tumors show distinct clustering with hypermethylation of tumor suppressor genes occurring more frequently than in pediatric cases. Enhanced PI3K/AKT pathway activation occurs in 45% of AYA cases versus 25% in pediatric tumors, potentially explaining differential treatment responses. The immune microenvironment demonstrates reduced infiltration compared to adult cases but increased compared to pediatric tumors, suggesting potential immunotherapy opportunities [14].

While Groups 3 and 4 predominate in pediatric cohorts, SHH-subgroup tumors occur with increased frequency among AYAs, commonly harboring PTCH1 mutations and demonstrating sonic hedgehog pathway hyperactivation (Table 1) [15].

Molecular profiling has emerged as a cornerstone of contemporary risk assessment, facilitating individualized treatment selection that optimizes efficacy while minimizing toxicity [16]. This precision medicine approach assumes particular importance for AYAs, whose tumors exhibit unique biological characteristics during this developmental transition period.

## 3. Evolution of Pediatric Treatment Protocols

Pediatric medulloblastoma therapy has undergone substantial refinement since initial chemotherapeutic approaches emerged in the 1960s, incorporating vincristine, methotrexate, and nitrosourea compounds. Subsequent key developmental milestones include the 1960s–1970s introduction of vincristine, methotrexate, and nitrosourea compounds; the 1980s–1990s establishment of cooperative group frameworks (SIOP, CCG); the 2000s–2010s integration of molecular subgrouping and risk stratification; and the 2010s-present development of precision medicine approaches with targeted therapies

Successive cooperative group studies led by international pediatric oncology organizations, including SIOP and CCG, established fundamental treatment frameworks [17,18]. Pioneering trials such as SIOP1 and CCG942 evaluated craniospinal irradiation monotherapy versus combined modality approaches [19]. Although demonstrating limited overall advantages, these investigations identified benefits for patients presenting with extensive disease burden [20].

The incorporation of platinum-containing regimens marked a pivotal advancement in survival outcomes. Contemporary treatment strategies are age-stratified: children under 3 years typically receive chemotherapy-only protocols to prevent severe neurological, cognitive, and endocrine complications associated with radiotherapy, while older children receive optimized chemotherapy combinations designed to minimize long-term organ dysfunction while maximizing therapeutic benefit [21,22,23]. North American protocols commonly employ cisplatin, cyclophosphamide, vincristine, and CCNU combinations, while European strategies frequently incorporate etoposide, carboplatin, ifosfamide, and high-dose methotrexate. The therapeutic role of autologous stem cell transplantation following high-dose chemotherapy in metastatic disease continues under active investigation [24,25,26].

Standard-risk medulloblastoma management (absence of metastases and residual disease <1.5 cm^2^) has achieved relative consensus. Radiotherapy initiation typically occurs within 4–5 weeks following neurosurgical intervention [27,28]. The landmark 9961 trial compared cisplatin/CCNU/vincristine with cisplatin/cyclophosphamide/vincristine regimens, identifying patient populations potentially benefiting from reduced radiotherapy doses and exploring craniospinal irradiation reduction from 36 Gy to 18–23.4 Gy in standard-risk cases [29,30]. Although the 9961 trial demonstrated equivalent mortality between treatment arms, it revealed significant ototoxicity associated with platinum agents, prompting subsequent protocol modifications. Recent approaches have focused on delivering six cycles of cisplatin/CCNU/vincristine alternating with three cycles of cyclophosphamide/vincristine [31,32].

Current trials continue investigating toxicity reduction strategies, including sodium thiosulfate addition (COG NCT05382338, currently suspended due to supply constraints). The SJMB96 and SJMB03 trials utilized intensive regimens combining vincristine, cisplatin, and cyclophosphamide followed by autologous stem cell transplantation after radiotherapy completion, achieving five-year progression-free survival exceeding 80% despite reduced craniospinal irradiation doses [33,34]. The ongoing SJMB12 trial (NCT01878617) emphasizes genetic risk stratification and novel therapeutic interventions, including Vismodegib maintenance for post-pubertal SHH-subgroup patients and gemcitabine-pemetrexed combinations for high-risk disease [35].

The SIOP PNET-5 protocol represents a contemporary multinational framework for medulloblastoma management in pediatric populations, incorporating molecular subgrouping alongside traditional clinical parameters. This collaborative approach addresses the heterogeneous nature of medulloblastoma through refined risk stratification that integrates histological variants, molecular signatures including WNT, SHH, Group 3, and Group 4 classifications, extent of surgical resection, and metastatic status. Treatment allocation follows a tripartite risk model where standard-risk patients receive reduced-intensity therapy to minimize long-term sequelae, while high-risk cases undergo intensified multimodal treatment incorporating maximal safe resection, risk-adapted craniospinal irradiation, and combination chemotherapy regimens featuring carboplatin, cyclophosphamide, and vincristine. The protocol emphasizes the critical role of complete surgical resection when feasible, as residual disease significantly influences prognosis and subsequent therapeutic decisions. Radiation therapy considerations reflect contemporary understanding of age-related neurotoxicity, with craniospinal irradiation reserved for appropriate candidates based on molecular subgroup, dissemination status, and age thresholds [36].

Attempts to eliminate craniospinal irradiation in low-risk patients proved unsuccessful due to early disease progression. Also, concurrent chemoradiotherapy investigations have yielded variable results [37]. Etoposide administration during radiotherapy increased mucocutaneous toxicity, while carboplatin improved survival specifically in Group 3 medulloblastoma [38,39]. A North American trial examining high-dose chemotherapy with autologous stem cell transplantation was terminated due to hepatic veno-occlusive disease, whereas a Milan-based pilot study of hyperfractionated accelerated radiotherapy preceded by intensive chemotherapy achieved 72% five-year overall survival with acceptable toxicity profiles [37,40,41].

A French trial, subsequently adopted across Europe, demonstrated 76% five-year overall survival using neoadjuvant carboplatin/etoposide followed by high-dose chemotherapy with autologous stem cell transplantation and risk-adapted radiotherapy [38]. The German multicenter HIT2000 study reported 74% five-year event-free survival employing induction chemotherapy (cyclophosphamide, vincristine, high-dose methotrexate, carboplatin, etoposide), intraventricular methotrexate, hyperfractionated radiotherapy, and maintenance chemotherapy [38,42,43].

## 4. Adult-Focused Treatment Approaches

Adult medulloblastoma management has predominantly relied on extrapolation from pediatric experience given disease rarity. Standard care involves maximal safe surgical resection targeting residual tumor volume < 1.5 cm^2^ while preserving essential neurological function.

Post-operative management typically includes craniospinal radiotherapy (36 Gy) with posterior cranial fossa boost (18–19.8 Gy) achieving cumulative doses of 54–55.8 Gy [44,45]. Advanced radiation techniques including intensity-modulated radiotherapy, volumetric-modulated arc therapy, and helical tomotherapy, optimize treatment precision. Radiotherapy initiation within four weeks post-surgery correlates with improved outcomes [45,46,47].

While adults experience reduced neurocognitive sequelae from radiotherapy compared to children, they frequently encounter increased chemotherapy-related toxicity. Optimal chemotherapy regimen selection and sequencing remain controversial in adult medulloblastoma [48]. The Packer regimen (vincristine, CCNU, cisplatin), adapted from pediatric protocols, represents a commonly utilized approach. The HIT-2000 trial demonstrated 68% four-year event-free survival and 89% overall survival in non-metastatic adult medulloblastoma using this regimen with radiotherapy. Given increased ototoxicity risk, carboplatin substitution for cisplatin has been proposed, particularly in neoadjuvant settings, though prospective adult validation remains incomplete [48,49,50].

Beyond auditory complications, adult patients frequently experience prolonged leukopenia and vincristine-induced peripheral neuropathy [51]. Meta-analyses demonstrate survival benefits for combined chemoradiotherapy versus radiotherapy alone. A comprehensive analysis of 907 patients across 227 studies revealed median survival of 108 months with chemotherapy versus 29 months with radiotherapy monotherapy [52].

A recent SEER database analysis of 333 adult medulloblastoma patients (2010–2018) demonstrated significantly improved cancer-specific survival with chemotherapy regardless of sequencing relative to radiotherapy (five-year CSS: 89% versus 72%, *p* = 0.038) [53]. Current evidence supports chemotherapy inclusion for improved survival outcomes in adults, consistent with pediatric observations.

Based on available evidence, European Association of Neuro-Oncology and EURACAN guidelines recommend incorporating chemotherapy for all adult medulloblastoma patients regardless of risk stratification. Treatment should occur in specialized centers with multidisciplinary teams including pediatric oncologists, neurosurgeons, radiologists, radiation oncologists, neurologists, and psychologists [54].

Without definitive randomized trials establishing superiority of specific chemotherapeutic regimens, treatment decisions require collaborative multidisciplinary assessment of systemic comorbidities, concurrent medications, age, and medulloblastoma risk classification. Vigilance regarding increased adult toxicity profiles remains essential, and investigational therapies warrant consideration for refractory or recurrent disease [50,55,56].

## 5. Contemporary Research Initiatives and Clinical Trials

Long-term medulloblastoma survival has improved dramatically from 20% in the 1950s to exceeding 80% currently, attributable to integrated multimodal therapy, surgical and radiotherapeutic advances, treatment intensification, and enhanced supportive care [57,58].

For pediatric patients aged 3–5 years and adolescents with newly diagnosed medulloblastoma, management universally combines maximal safe resection, craniospinal radiotherapy with posterior fossa boost, and chemotherapy. Standard radiotherapy protocols begin at 36 Gy, escalating to 54–55.8 Gy for the posterior fossa [57,58,59]. The COG A9961 trial demonstrated equivalent efficacy with reduced craniospinal irradiation (23.5 Gy versus 36 Gy), achieving greater than 80% five-year event-free survival [59].

The COG ACNS00331 trial investigated further dose reduction to 18 Gy, revealing inferior event-free and overall survival, particularly affecting Groups 3 and 4, while WNT-subgroup patients maintained favorable outcomes [60,61]. This underscores the importance of molecular subtyping in treatment planning. Protocols including SJMB-96 and SJMB-03 confirmed efficacy of reduced craniospinal irradiation followed by intensive chemotherapy [62].

Adult medulloblastoma presents distinct clinical characteristics and therapeutic challenges. Management typically combines surgery with craniospinal radiotherapy, with variable adjuvant chemotherapy approaches [63,64]. Evidence regarding chemotherapy’s prognostic impact remains inconsistent, with some studies demonstrating improved progression-free and overall survival while others show no benefit. Adverse effects frequently necessitate dose reductions or treatment discontinuation in adults [63,64,65]. Emerging adult medulloblastoma trials such as EORTC 1634-BTG/NOA-23 and AMBUSH aim to refine treatment through randomized assessment of radiotherapy protocols and targeted therapies for specific molecular subgroups, particularly SHH-subgroup tumors. These studies emphasize multidisciplinary collaboration incorporating pediatric oncology expertise [66,67,68,69].

Contemporary clinical trials investigating novel therapeutic agents represent a paradigm shift toward precision medicine approaches in medulloblastoma management. Several innovative drug classes are currently under investigation across multiple phases of clinical development.

Hedgehog pathway inhibitors constitute a major focus for SHH-subgroup medulloblastoma, particularly relevant for AYA populations where this subgroup predominates. Vismodegib (GDC-0449), a first-generation smoothened antagonist, has demonstrated activity in recurrent SHH medulloblastoma, leading to its investigation in maintenance therapy within the SJMB12 trial (NCT01878617) for post-pubertal patients. Second-generation agents including sonidegib (LDE225) and glasdegib (PF-04449913) are being evaluated in phase I/II studies, offering improved CNS penetration and reduced resistance potential [70,71].

Immunotherapy approaches are gaining attention with checkpoint inhibitors showing promise in select cases. Nivolumab and pembrolizumab are under investigation in recurrent medulloblastoma through basket trials, while CAR-T cell therapies targeting B7-H3 and other tumor-associated antigens are in early-phase development. The PNOC015 trial (NCT04185038) specifically evaluates personalized neoantigen vaccines in pediatric brain tumors, including medulloblastoma [72,73].

Epigenetic modulators represent another promising therapeutic avenue. Histone deacetylase inhibitors including vorinostat and panobinostat are being evaluated in combination with conventional therapy. DNA methyltransferase inhibitors such as 5-azacytidine show preclinical activity, particularly in Group 3 and Group 4 subtypes. The ongoing PNOC017 trial (NCT03416530) investigates combination epigenetic therapy with radiotherapy [74,75].

Protein degradation targeting through proteolysis-targeting chimeras (PROTACs) and molecular glue degraders represents an emerging therapeutic modality. These agents target previously “undruggable” proteins including MYC, which plays crucial roles in Group 3 medulloblastoma pathogenesis. Several compounds are advancing toward clinical evaluation [76,77].

Radiopharmaceutical approaches utilizing targeted alpha therapy and peptide receptor radionuclide therapy are being explored for leptomeningeal disease. These approaches may offer improved efficacy for disseminated disease while reducing systemic toxicity compared to conventional chemotherapy [78].

## 6. Unique Challenges in AYA Management

Medulloblastoma management in AYAs presents distinctive challenges including delayed diagnosis, unique biological tumor characteristics, and substantial treatment-related morbidity [79,80]. AYAs typically present with more advanced disease and remain underrepresented in clinical trials, limiting access to tailored therapies. Comprehensive survivorship care and dedicated AYA programs are essential to address their specific requirements and optimize long-term outcomes [81,82,83].

### 6.1. Clinical Presentation and Diagnostic Delays

AYA medulloblastoma presents distinct diagnostic challenges: classic pediatric symptoms (ataxia, hydrocephalus) occur in only 60% of AYA cases versus 85% in children; median time to diagnosis is 4.2 months in AYAs versus 2.1 months in children; 35% of AYAs present with metastatic disease versus 25% in pediatric cases.

Prognostic factors demonstrate consistency across pediatric and adult populations, facilitating risk stratification, though limited adult cohort data necessitates cautious interpretation [10,44,80,84]. Histological subtypes show age-dependent variations, with desmoplastic histology occurring more frequently in adults [15,85,86]. Lateral localization and hemispheric involvement also occur more commonly in adults [87].

Overall survival differences between children and adults appear minimal. However, late relapses occur more frequently in adults, typically 5–10 years post-treatment—up to 20% experience relapse after five years [45,88,89,90]. While extracranial metastases occur less frequently in adults, they more commonly affect bones and lungs, whereas hepatic metastases predominate in children [84,91].

AYAs often experience diagnostic delays due to medulloblastoma’s rarity in adults and nonspecific symptomatology [92]. Classic symptoms such as ataxia and hydrocephalus occur less frequently in AYAs compared to younger children. These diagnostic delays frequently result in more advanced disease at presentation, necessitating more aggressive treatment and yielding inferior survival outcomes [80,93,94].

### 6.2. Molecular and Biological Distinctions

The molecular landscape of AYA medulloblastoma demonstrates distinct characteristics that fundamentally differ from pediatric counterparts, necessitating specialized therapeutic approaches. Comprehensive genomic profiling reveals subgroup-specific alterations that influence both prognosis and treatment selection.

SHH Pathway Alterations: The SHH subgroup, predominant in AYAs, harbors diverse genetic alterations affecting pathway components. PTCH1 mutations occur in approximately 40–50% of AYA SHH tumors, compared to 20–30% in pediatric cases. These mutations result in constitutive pathway activation through loss of negative regulation. SUFU mutations, though less common (10–15%), demonstrate particular therapeutic relevance as they confer sensitivity to smoothened antagonists. SMO mutations, occurring in 5–10% of cases, may predict resistance to first-generation hedgehog inhibitors [95].

TP53 Pathway Disruption: TP53 mutations occur with increased frequency in AYA medulloblastoma (15–20%) compared to pediatric populations (5–10%), particularly within SHH subgroups. These mutations often co-occur with PTCH1 alterations and associate with inferior prognosis and increased risk of secondary malignancies following radiotherapy. The presence of TP53 mutations influences treatment decisions, with some protocols recommending reduced radiation doses to minimize long-term cancer risk [96,97].

Chromatin Remodeling Gene Alterations: AYA medulloblastomas demonstrate distinct patterns of chromatin remodeling gene mutations. CREBBP and EP300 mutations occur in 20–25% of cases, affecting histone acetylation and transcriptional regulation. These alterations create therapeutic opportunities for histone deacetylase inhibitor combinations. Additionally, mutations in SWI/SNF complex components (ARID1A, ARID2, SMARCA4) occur in 10–15% of AYA cases, potentially conferring sensitivity to synthetic lethal approaches targeting DNA repair pathways [98,99].

DNA Repair Pathway Defects: Homologous recombination deficiency occurs more frequently in AYA medulloblastoma, with BRCA1/BRCA2 pathway alterations identified in 8–12% of cases. These defects create sensitivity to PARP inhibitors and platinum-based chemotherapy, offering personalized treatment opportunities. Additionally, mismatch repair deficiency, while rare (2–3%), may predict immunotherapy responsiveness through increased tumor mutational burden [100].

Telomerase and Cell Cycle Regulation: TERT promoter mutations occur in 15–20% of AYA medulloblastomas, particularly in SHH subgroups, contributing to cellular immortalization. These alterations associate with improved survival outcomes, potentially reflecting enhanced treatment sensitivity. Cell cycle regulatory gene alterations, including CDKN2A/2B deletions and RB1 mutations, occur in 25–30% of cases and may predict CDK4/6 inhibitor sensitivity [101,102].

Metabolic Reprogramming: AYA medulloblastomas demonstrate distinct metabolic profiles characterized by enhanced glycolysis and altered amino acid metabolism. IDH1/IDH2 mutations, while uncommon (2–5%), create opportunities for targeted metabolic interventions. Additionally, alterations in mTOR pathway components (PIK3CA, AKT, mTOR) occur in 20–25% of cases, suggesting potential benefit from mTOR inhibitor combinations [103,104].

The higher incidence of targetable genetic alterations in AYA medulloblastoma provides opportunities for precision medicine approaches, though clinical validation through dedicated trials remains necessary (Table 2) [105,106,107].

Unfortunately, development of personalized approaches remains hindered by the absence of large-scale AYA-specific clinical trials [82,104,105,106,107,108]. These biological disparities contribute to variations in prognosis and treatment response, potentially reducing conventional therapeutic efficacy. This necessitates customized treatment strategies acknowledging AYAs’ unique characteristics. Understanding molecular variations remains critical for developing tailored therapeutic approaches and improving outcomes in this distinctive population [106,109,110,111].

### 6.3. Treatment-Related Toxicity and Long-Term Sequelae

Treatment-related toxicity represents a major challenge in AYA medulloblastoma management [112]. Chemotherapy, particularly platinum-based regimens, can cause severe ototoxicity, nephrotoxicity, and hematological toxicity [113,114]. AYAs demonstrate particular vulnerability to adverse effects due to their age and frequent treatment with pediatric-derived regimens, increasing risks of long-term sequelae including hearing loss, cognitive deficits, infertility, and secondary malignancies [115,116].

Emerging protocols are evolving in response, modifying chemotherapy approaches, limiting craniospinal irradiation in selected cases, and exploring innovative therapies including targeted agents and immunotherapy [116,117,118]. Nevertheless, conventional treatment toxicity remains a substantial barrier to optimal AYA care, resulting in significant long-term effects and quality of life impairment. This underscores the urgent need for developing more targeted, less toxic therapeutic options [28,119,120,121,122].

### 6.4. Clinical Trial Access and Participation Barriers

AYA underrepresentation in clinical trials significantly impedes outcome improvement. Trial designs frequently impose age restrictions excluding AYAs or fail to address their unique requirements. This participation deficit has contributed to stagnant survival rates for various tumor types in this age group, leaving AYAs positioned between pediatric and adult oncology paradigms [82,123,124,125,126].

Research demonstrates AYA patients with specific tumor types, including acute lymphoblastic leukemia and sarcomas, derive significant benefit from pediatric treatment protocols with superior survival outcomes [127,128]. Unfortunately, AYAs with medulloblastoma typically cannot access pediatric protocols due to exclusion from studies designed for younger patients. Improving AYA trial participation requires developing inclusive, age-appropriate study designs [81,129].

Enhancing clinical trial enrollment necessitates multi-center collaborations and revised eligibility criteria accommodating AYAs. “Opt-out” enrollment models—automatically enrolling patients unless they withdraw—may increase participation in AYA-targeted trials. Limited participation restricts understanding of optimal AYA-specific protocols, leaving unique biological and psychosocial factors inadequately addressed [130,131,132,133].

### 6.5. Survivorship Care and Care Transition

Survivorship care represents a crucial component of comprehensive AYA medulloblastoma management. Long-term survivors frequently encounter neurocognitive and physical challenges alongside psychosocial difficulties during reintegration into social and professional environments. The transition from pediatric to adult care presents vulnerability, often characterized by care discontinuity and insufficient specialized support [134,135,136,137].

**Figure 1:**
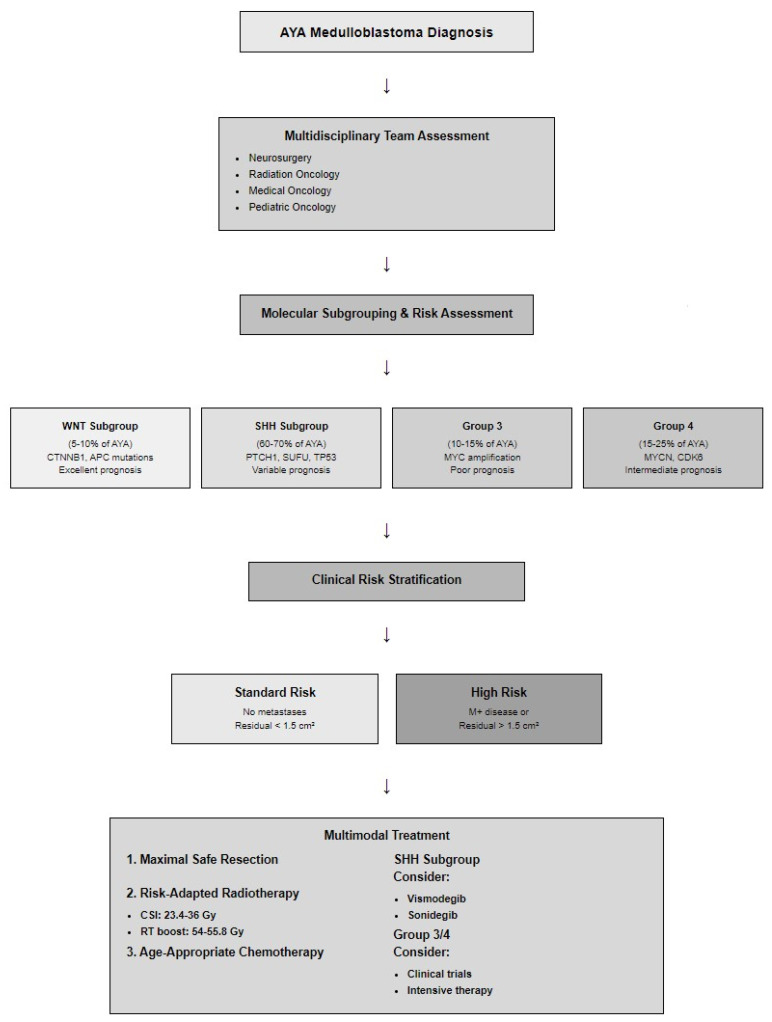
Management algorithm for medulloblastoma in AYA patients.

Dedicated AYA programs have emerged as a key strategy addressing these challenges [83,138]. These programs provide comprehensive, patient-centered care addressing medical, emotional, and psychosocial needs. By integrating pediatric and adult oncology expertise, AYA programs deliver tailored care reflecting specific tumor biology and developmental stage (Figure 1) [139,140].

Research on AYA program establishment emphasizes early collaboration between pediatric and adult oncology teams during planning phases, ensuring meaningful contribution from all stakeholders. AYA advocates play essential roles fostering collaboration and enhancing program effectiveness. Medical and political sector participation remains crucial for strengthening AYA communities at local, national, and international levels to support these initiatives [141,142,143,144].

## 7. Enhancing Pediatric–Adult Oncology Collaboration

Effective AYA care requires systematic collaboration between pediatric and adult oncology teams through joint clinics with combined pediatric–adult consultations; shared protocols and standardized treatment algorithms; regular multidisciplinary conferences; cross-specialty training programs; pooled databases and outcome registries; and joint trial design with age-inclusive protocols spanning 15–39 years.

Insufficient collaboration between pediatric and adult oncology teams represents a significant obstacle to effective AYA program implementation. Institutional competition regarding AYA patient management has been documented in various studies, undermining efforts to create specialized programs delivering optimal care [145,146,147,148]. Given the diverse brain tumor spectrum encountered in this population, typically managed by either pediatric or adult specialists, robust inter-team collaboration remains essential for optimal outcomes [149].

Despite AYAs’ unique characteristics, international treatment guidelines and clinical trials predominantly target either pediatric or adult populations, substantially improving survival and reducing treatment-related toxicity in these groups. AYAs remain underrepresented in clinical trials, with many excluding participants over age 18, limiting access to age-appropriate treatment options (Table 3).

Moreover, substantial numbers of AYA patients receive care in general hospitals rather than specialized cancer centers with appropriate expertise [82,123,150,151]. In addition, the management of recurrent medulloblastoma in AYA presents unique therapeutic challenges, as delayed recurrences are more frequent in this population compared to pediatric cases, often occurring beyond the timeframe when high-dose chemotherapy with stem cell rescue remains feasible. Contemporary salvage strategies must account for the cumulative toxicity burden from prior treatments, the potential for secondary malignancies, and the need to preserve quality of life in patients with longer life expectancy than their pediatric counterparts.

Dedicated AYA programs have become increasingly important for addressing specific physical and psychosocial needs, enhancing overall care quality [138,152]. Recent guidelines support program establishment in the United States. Current oncology care models, including family-centered pediatric approaches, often inadequately address AYAs’ unique needs, creating a care gap. AYA programs provide specialized, age-appropriate care and comprehensive support encompassing clinical and psychosocial dimensions [153,154,155].

Strengthening the AYA community across local, national, and international levels remains essential for garnering political and medical sector support, enhancing healthcare professional education, and developing comprehensive guidelines [144,156]. Research demonstrates effective systems and coordination improve patient satisfaction through age-appropriate information and specialized services. Significant progress has occurred in AYA treatment, exemplified by the 2021 European Society for Medical Oncology and International Society of Pediatric Oncology position paper emphasizing collaboration importance [157,158]. The literature consistently highlights inconsistent AYA age definitions, reflecting ongoing discussion regarding appropriate age parameters [159]. The ESMO/SIOPE position paper recommended standardizing the 15–39 year age range to unify clinical care and research efforts [158].

Establishing clear referral and transition pathways between pediatric and adult oncology services enhances collaboration and improves care coordination. This underscores the necessity of ensuring AYA patients receive tailored care addressing their specific needs, regardless of treatment setting [160,161].

## 8. Conclusions

Future research must prioritize specific areas such as AYA-specific clinical trials with inclusive age criteria and molecular stratification; biomarker development for personalized treatment selection; toxicity reduction strategies through age-appropriate protocol modifications; survivorship research addressing long-term quality of life and late effects; and the establishment of international AYA medulloblastoma consortiums. However, international collaboration in rare CNS tumor research remains hampered by heterogeneous regulatory landscapes and institutional frameworks, despite the clear imperative for unified protocols to achieve adequate statistical power in diseases with limited patient populations worldwide.

Dedicated AYA programs emerge as fundamental requirements, providing multidisciplinary frameworks to address the complex interplay of medical, psychosocial, and developmental factors unique to this population. These programs must bridge the divide between pediatric and adult oncology, fostering collaboration that leverages expertise from both subspecialties while maintaining AYA-specific focus.

Survivorship care represents an equally critical component, as AYA survivors face decades beyond their diagnosis with treatment-related effects that may manifest years after therapy completion. The integration of advanced molecular profiling, innovative therapeutic agents, and precision medicine approaches holds promise for transforming outcomes, but requires sustained commitment to AYA-focused research and enhanced clinical trial participation.

Ultimately, optimal AYA medulloblastoma management requires creating a new paradigm that synthesizes the best elements of pediatric and adult approaches while addressing the distinct characteristics that define this population caught between two worlds of oncology care.

## Figures and Tables

**Table 1:** Molecular subgroup distribution and characteristics in AYA medulloblastoma.

Subgroup	AYA Frequency (%)	Key Genetic Alterations	Therapeutic Targets	Prognosis
WNT	5–10	CTNNB1, APC, SMARCA4	β-catenin inhibitors	Excellent
SHH	60–70	PTCH1, SUFU, SMO, TP53	Hedgehog inhibitors	Intermediate
Group 3	10–15	MYC, SMARCA4, KBTBD4	BET inhibitors, PROTACs	Poor-Intermediate
Group 4	15–25	MYCN, CDK6, SNCAIP	CDK inhibitors	Intermediate

CTNNB1: Catenin Beta 1; APC: Adenomatous Polyposis Coli; SMARCA4: SWI/SNF Related, Matrix Associated, Actin Dependent Regulator Of Chromatin, Subfamily A, Member 4; BET: Bromodomain and Extra-Terminal domain; PTCH1: Patched 1; SUFU: Suppressor Of Fused Homolog; SMO: Smoothened, Frizzled Class Receptor; TP53: Tumor Protein P53; MYC: MYC Proto-Oncogene; KBTBD4: Kelch Repeat And BTB Domain Containing 4; CDK6: Cyclin Dependent Kinase 6; SNCAIP: Synuclein Alpha Interacting Protein.

**Table 2:** Current clinical trials investigating novel agents in medulloblastoma.

Trial	Phase	Population	Intervention	Primary Endpoint
SJMB12	II	Pediatric/AYA	Vismodegib maintenance	Event-free survival
PNOC015	I/II	Pediatric	Neoantigen vaccines	Safety/Immunogenicity
PNOC017	I	Pediatric	Epigenetic therapy	Maximum tolerated dose
EORTC-1634	III	Adult	Radiotherapy optimization	Overall survival
AMBUSH	II	Adult SHH	Sonidegib + radiotherapy	Progression-free survival

**Table 3:** Clinical trials in medulloblastoma by age group.

Trial	Age Range	Design	Key Findings	AYA Relevance
**Pediatric Trials**				
COG A9961	3–21 years	Randomized Phase III	Reduced CSI non-inferior (23.4 vs. 36 Gy), >80% 5-year EFS	Moderate—Upper age included some AYAs
SJMB12	3–21 years	Phase II	Molecular stratification improves outcomes	High—Includes adolescents, targeted therapy for SHH
SIOP PNET-5	3–18 years	Phase III	Risk-adapted therapy based on molecular subgroups	Limited—Excludes most AYAs
**Adult Trials**				
HIT-2000	>18 years	Retrospective	68% 4-year EFS with pediatric-derived protocols	High—Direct relevance to young adults
EORTC 1634	>18 years	Phase II/III	Ongoing molecular stratification study	High—Includes AYA population
**AYA-Focused Trials**				
AMBUSH	16–65 years	Phase II	Targeting SHH subgroup with SMO inhibitors	Very High—AYA-specific design

## Abbreviations

The following abbreviations are used in this manuscript:

ACNS	A Children’s Oncology Group study designation
AMBUSH	Adult Medulloblastoma Study
AYA	Adolescents and Young Adults
BTG	Brain Tumor Group
CCG	Children’s Cancer Group
CNS	Central Nervous System
COG	Children’s Oncology Group
CSS	Cancer-Specific Survival
EORTC	European Organisation for Research and Treatment of Cancer
ESMO	European Society for Medical Oncology
EURACAN	European Reference Network for Rare Adult Solid Cancers
Gy	Gray
HIT	Hirntumor (German brain tumor study group)
NCT	National Clinical Trial
NOA	Neuro-oncology Working Group
